# The myopathy-causing mutation DNM2-S619L leads to defective tubulation *in vitro* and in developing zebrafish

**DOI:** 10.1242/dmm.012286

**Published:** 2013-10-17

**Authors:** Elizabeth M. Gibbs, Ann E. Davidson, William R. Telfer, Eva L. Feldman, James J. Dowling

**Affiliations:** 1 Department of Neuroscience, University of Michigan Medical Center, Ann Arbor, MI 48109-2200, USA.; 2 Department of Neurology, University of Michigan Medical Center, Ann Arbor, MI 48109-2200, USA.; 3 Department of Pediatrics, University of Michigan Medical Center, Ann Arbor, MI 48109-2200, USA.; 4 Department of Neurology, Hospital for Sick Children, Toronto, ON M5G 1×8, Canada.; 5 Department of Genetics and Genome Biology, Hospital for Sick Children, Toronto, ON M5G 1×8, Canada.; 6 Department of Paediatrics, University of Toronto, Toronto, ON M5G 0A4, Canada.; 7 Department of Molecular Genetics, University of Toronto, Toronto, ON M5G 0A4, Canada.

**Keywords:** Dynamin-2, Excitation-contraction coupling, Myopathy

## Abstract

DNM2 is a ubiquitously expressed GTPase that regulates multiple subcellular processes. Mutations in *DNM2* are a common cause of centronuclear myopathy, a severe disorder characterized by altered skeletal muscle structure and function. The precise mechanisms underlying disease-associated *DNM2* mutations are unresolved. We examined the common DNM2-S619L mutation using both *in vitro* and *in vivo* approaches. Expression of DNM2-S619L in zebrafish led to the accumulation of aberrant vesicular structures and to defective excitation-contraction coupling. Expression of DNM2-S619L in COS7 cells resulted in defective BIN1-dependent tubule formation. These data suggest that DNM2-S619L causes disease, in part, by interfering with membrane tubulation.

## INTRODUCTION

Centronuclear myopathies are a clinically and genetically heterogeneous group of skeletal muscle disorders that share common muscle biopsy features. These features include myofiber hypotrophy, an increased number of centrally located myonuclei, and irregularities in oxidative stains. Recent evidence suggests that another unifying feature of centronuclear myopathies is abnormalities in triad structure (Al-Qusairi et al., 2009; Dowling et al., 2009; Nicot et al., 2007; Toussaint et al., 2011). The triad represents the intersection between the T-tubule and the terminal sarcoplasmic reticulum, and is a key component of the cellular apparatus that mediates excitation-contraction coupling. Changes in the structure of the triad have been observed in all genetically confirmed subtypes of centronuclear myopathy (Dowling et al., 2009; Nicot et al., 2007; Toussaint et al., 2011).

Mutations in dynamin-2 (DNM2) are the most common cause of autosomal-dominant centronuclear myopathy (Hanisch et al., 2011; Jeub et al., 2008). They are also a rare cause of inherited peripheral neuropathy (Züchner et al., 2005). DNM2 is a large GTPase that is a member of the dynamin family of membrane fission proteins. Numerous functions, including the regulation of endocytosis, cell migration and cytokinesis, are associated with the normal function of DNM2 (Jones et al., 1998; McNiven et al., 2000; Thompson et al., 2004). The mechanism(s) via which dominant mutations in *DNM2* cause myopathy, however, are incompletely understood. Data from cell culture models has been conflicting; for example, some studies have identified abnormalities in endocytosis, whereas others have not (Bitoun et al., 2009; Koutsopoulos et al., 2011; Liu et al., 2011; Tanabe and Takei, 2009). Examination by immunohistochemistry of patient muscle biopsies revealed disorganization of the triad, and recent studies using viral expression in the mouse corroborated this observation (Cowling et al., 2011). However, the mechanism(s) by which *DNM2* mutations impair triad formation is not known; furthermore, a functional effect on excitation-contraction coupling has yet to be documented.

In this study, we examine the S619L mutation in the pleckstrin homology domain of DNM2. The S619L mutation is one of the most common DNM2 mutations and is associated with a severe centronuclear myopathy muscle phenotype (Bitoun et al., 2007; Böhm et al., 2012). We determined the impact of DNM2-S619L expression in both the developing zebrafish and in a cell culture model of tubule formation (Bitoun et al., 2007). We show that the mutation results in defective membrane tubulation, and propose that one mechanism of disease is the interference of BIN1-mediated tubule formation and subsequent impairment of excitation-contraction coupling.

## RESULTS AND DISCUSSION

### Expression of DNM2-S619L in developing zebrafish disrupts triad structure

To generate a model of centronuclear myopathy due to *DNM2* mutation, we ubiquitously expressed either wild-type (DNM2-WT) or S619L-mutation-containing (DNM2-S619L) human *DNM2* RNA in the developing zebrafish starting at the one- to two-cell stage (supplementary material Fig. S1). Expression of DNM2-S619L results in abnormalities in muscle structure (including abnormally located nuclei and perinuclear disorganization), impaired force generation and altered motor function. The general phenotypic features of DNM2-S619L zebrafish are recently published (Gibbs et al., 2013). In this study we specifically focused on the triad because of the mounting data suggesting that triad defects are a crucial aspect of centronuclear myopathy (Al-Qusairi et al., 2009; Dowling et al., 2009; Nicot et al., 2007; Toussaint et al., 2011). We performed ultrastructural evaluation of the triad structure in zebrafish with confirmed expression of either DNM2-WT or DNM2-S619L.

**Fig. 1.**
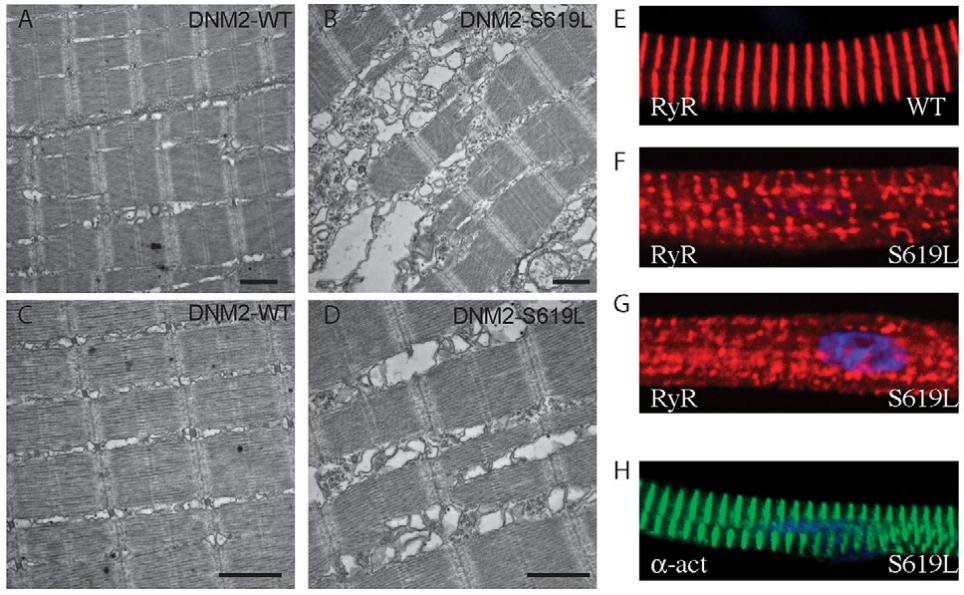
**T-tubule and sarcoplasmic reticulum abnormalities in DNM2-S619L larval muscle.** (A–D) Electron micrographs of longitudinal sections through zebrafish muscle at 3 dpf. (A,C) Muscle from DNM2-WT larvae shows the typical sarcomere striations of vertebrate striated muscle. (B,D) Muscle from DNM2-S619L larvae demonstrates extensive swelling and vacuolization in the region of the SR and T-tubules. Scale bars: 1 μm. (E-H) Confocal micrographs of isolated myofibers subjected to immunofluorescence analysis. (E) Wild-type (WT) myofiber showing the expected pattern of RyR1 staining. (F,G) RyR1 expression is irregular in DNM2-S619L myofibers, and is often found aggregated. (H) α-actinin staining was normal, indicating that other elements of the muscle structure in S619L myofibers are not disturbed.

Electron micrographs of skeletal muscle from three separate DNM2-WT or DNM2-S619L zebrafish, obtained at 3 days post fertilization (dpf), are depicted in Fig. 1. DNM2-WT muscle showed a normal appearance and distribution of T-tubule and terminal sarcoplasmic reticulum (Fig. 1A,C). Conversely, muscle from DNM2-S619L larvae had extensive triad abnormalities (Fig. 1B,D). Although there were rare regions of normal triads, the majority did not resemble the native structure but were instead characterized by disorganized and swollen membrane structures. The most striking aspect of the triad defects was the abundant accumulation of large, irregular vesicular structures that correspond in location and by general appearance to the terminal sarcoplasmic reticulum. Overall, these findings reflect substantial membrane abnormalities in the muscle of DNM2-S619L zebrafish.

**TRANSLATIONAL IMPACT**

**Clinical issue**

Congenital myopathies are a heterogeneous group of childhood-onset muscle diseases associated with significant morbidity and early mortality. To date, no therapies are available for any congenital myopathy. Dominant mutations in *DNM2* (dynamin-2) are associated with a common congenital myopathy subtype called autosomal dominant centronuclear myopathy. Much remains to be understood regarding the pathogenesis of this condition, with one key barrier being the relative lack of adequate models of the disease. The goal of this study was to further characterize zebrafish and cell culture models of DNM2-related myopathy and to use these models to better understand disease pathogenesis and potentially inform the development of suitable therapies.

**Results**

The skeletal muscle phenotype of early-stage zebrafish embryos injected with wild-type or mutant *DNM2* RNA was characterized. Larvae expressing mutant DNM2 (DNM2-S619L) had structural abnormalities in the triad, the skeletal muscle substructure that represents the intersection of the T-tubule and terminal sarcoplasmic reticulum. Subsequent functional studies revealed that contraction-related calcium release was also defective in DNM2-S619L embryos. The authors also subjected the larvae to ultrastructural analysis, which, combined with the functional data, indicated a defect in excitation-contraction coupling. This led to a hypothesis that the DNM2-S619L mutation promotes defective membrane tubulation, which was tested using an *in vitro* tubulation assay. Exogenous expression of DNM2-S619L cDNA (but not wild-type DNM2) into COS7 cells altered the production of normal tubules, thus supporting the idea that *DNM2* mutation, at least in part, causes disease by preventing proper tubulation.

**Implications and future directions**

The data in this short report demonstrate that a common congenital-myopathy-associated mutation disturbs tubule formation, both *in vitro* and in a zebrafish model of the disease. In turn, this abnormal tubulation prevents normal excitation-contraction coupling, which likely leads to muscle weakness and impaired muscle force generation. This new knowledge of underlying disease pathogenesis addresses an unanswered aspect of DNM2-related myopathy, and will serve as a springboard for rational therapy development for this devastating childhood disorder and related congenital myopathies.

To support our assertion that the abnormal structures seen by electron microscopy are, in fact, triads, we performed immunofluorescent analysis of triad markers on myofibers isolated from RNA-injected embryos. Myofibers from uninjected and DNM2-WT-injected embryos demonstrated the expected pattern of RyR1 (Fig. 1E) and DHPR (data not shown) staining. Conversely, myofibers from DNM2-S619L zebrafish had widespread abnormalities in RyR1 and DHPR staining, including aggregation of staining and interruption of the normal confluent linear pattern (Fig. 1F,G). Of note, α-actinin staining (highlighting Z-bands) was normal (Fig. 1H), suggesting that the changes in triad marker immunofluorescence were not due to non-specific dissolution of the entire myofiber structure. In all, these data corroborate the ultrastructural analysis and demonstrate severe disturbance of the triad structure with DNM2-S619L expression.

### Impaired excitation-contraction coupling in the muscle of DNM2-S619L larvae

**Fig. 2.**
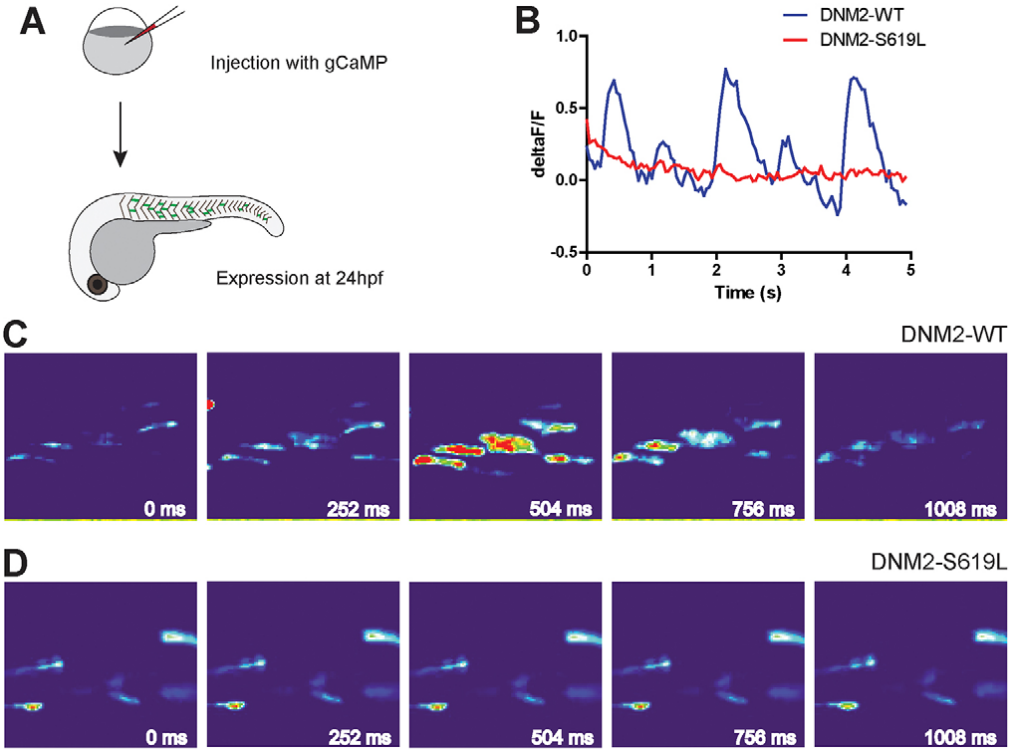
**Calcium activity in DNM2-S619L larvae muscle.** (A) Embryos were co-injected with DNA for *GCaMP* (a genetically encoded calcium indicator) and RNA for *DNM2* (WT or S619L) for GCaMP fluorescent imaging in muscle during spontaneous muscle contractions at 24 hpf. (B) Representative trace of whole-embryo fluorescent intensity (ΔF/F) during spontaneous contractions. Although DNM2-S619L larvae displayed spontaneous contractions with normal timing, there was no substantial increase in GCaMP fluorescence. (C,D) Sequential images of GCaMP fluorescence from the larvae in panel B.

The abnormalities in triads in our zebrafish model correspond well with the immunohistological abnormalities previously reported in DNM2 patient biopsies and the ultrastructural changes described in the viral overexpression model of DNM2-associated centronuclear myopathy (Cowling et al., 2011; Toussaint et al., 2011). The potential impact of structural triad changes on triad function, however, has yet to be examined for any DNM2 model. In order to determine the functional effect(s) of DNM2-S619L expression on excitation-contraction coupling, we examined calcium transients in spontaneously contracting embryos using a genetically encoded calcium indicator, GCaMP3. Embryos co-injected with *DNM2* RNA and *GCaMP3* cDNA were screened for variegated GCaMP expression in skeletal muscle (Fig. 2A). Both DNM2-WT and DNM2-S619L larvae expressed GCaMP3 at a similar level. At ~24 hours post fertilization (hpf), embryos were embedded in agarose and fluorescence was measured during spontaneous muscle contractions. Both DNM2-WT and DNM2-S619L embryos displayed abundant spontaneous muscle contractions at this point. In DNM2-WT embryos, a pulse of fluorescence corresponding to sarcoplasmic reticulum (SR)-based calcium release was temporally linked to each contraction. In DNM2-S619L embryos, however, there was minimal or no visible increase in fluorescence during muscle contraction in 10 of 12 embryos examined (Fig. 2C,D). In order to directly compare the fluorescence changes between embryos, we calculated the relative change in fluorescent recordings before versus during a muscle contraction (ΔF/F). We observed a significant difference in this dynamic ratio of calcium transients in DNM2-WT as compared with DNM2-S619L embryos (Fig. 2B and supplementary material Fig. S2). This indicated that, in addition to altering triad organization in zebrafish muscle, DNM2-S619L expression impairs stimulus-associated intracellular calcium release, a triad-mediated muscle function.

### DNM2-S619L expression alters the formation of BIN1-induced tubules

The above data reveal that DNM2-S619L expression can disrupt triad formation and function, but do not provide a potential mechanism via which it may do so. It has been previously established that DNM2-WT can interact with BIN1 (also called amphyphysin-2), a protein that can sense and regulate membrane curvature (Takei et al., 1999). A muscle-specific isoform of BIN1 (BIN1 isoform 8) has been shown to be required for T-tubule biogenesis, and recessive mutations in *BIN1* cause an autosomal-recessive form of centronuclear myopathy associated with triad defects (Lee et al., 2002; Nicot et al., 2007; Razzaq et al., 2001). Based on these known functions of BIN1, we hypothesized that DNM2-S619L mutations function to impair triad structure by interfering with BIN1-mediated tubulogenesis. To test this hypothesis, we used a well-established *in vitro* model of BIN1-dependent tubule formation (Nicot and Laporte, 2007; Nicot et al., 2007).

**Fig. 3.**
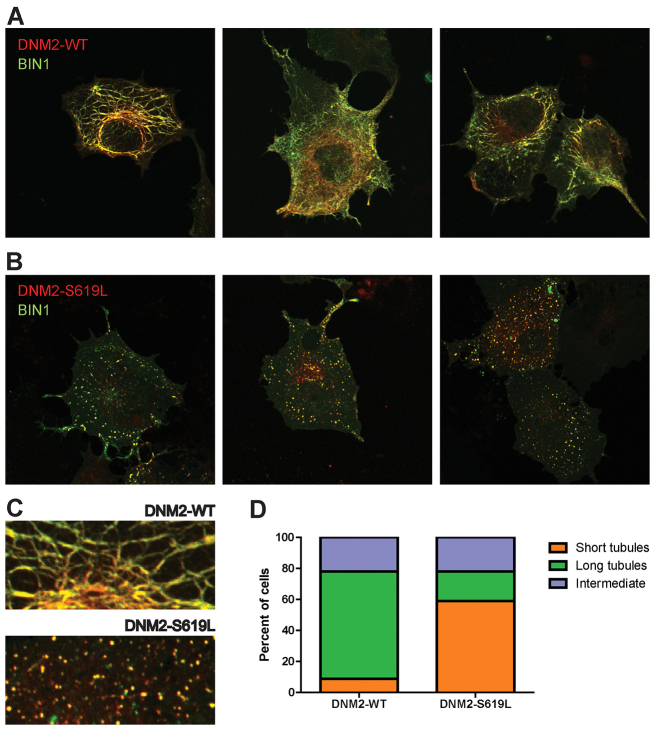
**DNM2-S619L expression alters *ex vivo* tubulation in COS7 cells.** (A) Co-expression of mCherry-DNM2-WT and GFP-BIN1. DNM2 and BIN1 colocalize to a complex membranous network of tubules induced by BIN1 overexpression. (B) Co-expression of BIN1 with DNM2-S619L disrupts the normal distribution of these networks. (C) Higher-magnification view of tubules from A and B, demonstrating long tubules (WT, top) and short/punctate tubules (S619L, bottom). (D) Quantification of tubulation in BIN1-induced cells overexpressing DNM2-WT versus DNM2-S619L. Whereas 69% of DNM2-WT-expressing cells exhibit long tubules, only 19% of DNM2-S619L-expressing cells have a long-tubule phenotype (DNM2-WT, *n*=68; DNM2-S619L, *n*=73; data combined from three independent experiments).

Expression of BIN1 isoform 8 (iso8) can induce the formation of tubular membrane structures when expressed in non-muscle cells, thus enabling an *in vitro* model of T-tubule biogenesis. To examine the impact of DNM2-S619L expression in this system, we co-expressed in COS7 cells GFP-tagged BIN1 iso8 and either DNM-WT cDNA or DNM2-S619L cDNA tagged with mCherry. Consistent with previously published results, we found by confocal microscopy that co-expression of BIN1 iso8 and DNM2-WT resulted in extensive tubulation (Fig. 3A). When DNM2-S619L was co-expressed with BIN1 iso8, however, we observed predominantly the formation of short punctate structures instead of tubules (Fig. 3B). To quantify these effects on membrane organization, tubulation in each cell was classified as short, long or intermediate. Whereas DNM2-WT-expressing cells exhibit a phenotype consisting of long tubules, only 19% of DNM2-S619L-expressing cells had a similar phenotype (Fig. 3D; DNM2-WT *n*=68, DNM2-S619L *n*=73, cells from three independent experiments). Thus, compared with DNM2-WT, expression of DNM2-S619L results in a significant impairment in BIN1-dependent tubule formation.

### Conclusions

We demonstrate that the common myopathy-causing S619L mutation in DNM2 disrupts triad structure and function *in vivo* and BIN1-induced tubulation *in vitro*. These data represent the first direct evidence that a DNM2 mutation can interfere with tubulation and disrupt triadic function. Based on these novel findings, we hypothesize that muscle disease related to DNM2 mutations is caused by the production of dominantly active defective DNM2 proteins that interfere with the tubulation process. Disrupted tubulation, in turn, impairs triad formation and excitation contraction, which results in abnormal muscle force generation and ultimately muscle weakness (Gibbs et al., 2013). Future experimentation, particularly with the testing of additional DNM2-associated centronuclear myopathy mutations, will be required to support this hypothesis. These data, however, provide an important step toward establishing the pathomechanisms related to DNM2 mutations.

## MATERIALS AND METHODS

### Animal care

Zebrafish (AB strain) were bred and raised according to established protocols approved by the University of Michigan Animal Care and Use Committee (UCUCA protocol #09835).

### Plasmid construction and RNA synthesis

Wild-type human *DNM2* plasmid was purchased from Invitrogen (ORF Gateway^®^ Entry IOH53617). The S619L point mutation was introduced using the QuikChange Lightning Site-directed Mutagenesis kit (Stratagene). Expression vectors were generated using the Gateway system and p5E-CMV/SP6, p3E-polyA and pDestTol2pA2 cassettes from the Tol2kit v1.2 (kind gift of Dr Chi-Bin Chien, University of Utah, UT). Gateway recombination reactions were performed using LR Clonase II Plus Enzyme Mix (Invitrogen). For RNA injection, plasmids were linearized with *Not*I and transcribed using the SP6 mMessage Machine kit (Ambion).

### RNA injection of zebrafish embryos

Zebrafish embryos were injected as previously described (Dowling et al., 2009). Briefly, fertilized eggs were injected at the one- to two-cell stage using a Nanoject II injector (Drummond Scientific). Embryos were injected with *DNM2* RNA (30 ng/μl) in a 4.6 nl volume.

### Muscle ultrastructure

Zebrafish at 3 dpf were fixed overnight in Karnovsky’s fixative and then processed for electron microscopy by the Microscopy and Imaging Laboratory (MIL) core facility at the University of Michigan. Electron microscopy was performed using a Phillips CM-100 transmission electron microscope.

### Myofiber immunofluorescence

Myofibers were isolated from 3-dpf zebrafish embryos using our established protocol (Horstick et al., 2013). Fibers were fixed to coverslips for 10 minutes in 4% paraformaldehyde. Immunostaining was done as previously reporting using antibodies to a-actinin (Sigma), RyR1 (Developmental Hybridoma Bank) and DHPR (Abcam) (Dowling et al., 2009; Majczenko et al., 2012).

### Measurements of calcium transients

The construct containing *GCaMP* under a muscle actin promoter was a kind gift of Dr John Kuwada (University of Michigan, MI). Plasmid DNA was co-injected with *DNM2* RNA at a concentration of 10 ng/μl in one- to two-cell embryos. At 24 hours, embryos were embedded and immobilized in 1% low-melt agarose. Fluorescent intensity during 10 seconds of spontaneous muscle contractions was recorded using a Nikon AZ-100 microscope. For image processing, ImageJ was used to measure total fluorescent intensity in a region of the larval trunk over the recorded interval. Baseline fluorescent levels were used to calculate relative fluorescence (AF/F).

### *Ex vivo* tubulation in COS cells

*Ex vivo* tubulation was performed following the basic methods described previously (Nicot et al., 2007). The BIN1 iso8-GFP construct was a kind gift of Dr Pietro De Camilli (Yale University, CT). COS7 cells were maintained in DMEM + 10% FBS and cultured on 22 mm^2^ coverslips. Cells were transfected using the *TransIT* reagent (Mirus). For processing, cells were fixed with 4% paraformaldehyde for 15 minutes, washed in PBS, and then mounted on Superfrost plus slides with ProLong plus DAPI. Images were generated using an Olympus confocal microscope and the FluoView software package. Tubulation patterns were classified as previously described (Meunier et al., 2009; Nicot et al., 2007). Cells with ‘short’ tubules exhibited punctate tubules with lengths no greater than four times the tubule width, whereas cells categorized as ‘long’ displayed tubules that extended uninterrupted to the plasma membrane. Cells with tubules longer than four times the width that did not fully extend to the plasma membrane were classified as ‘intermediate’.

### Statistical analysis

Statistical analysis was performed using the GraphPad Prism 5 software package. Significance was determined using Student’s *t*-test or Fisher’s exact test.

## Supplementary Material

Supplementary Material
