# Supplementary material for: The myopathy-causing mutation DNM2-S619L leads to defective tubulation in vitro and in developing zebrafish
Source: Dis Model Mech. 2013 Oct 17;7(1):157–61. doi: 10.1242/dmm.012286 (PMC3882057; doi:10.1242/dmm.012286)
Supplement: Supplementary Material [file supp_7_1_157__index.html]

The myopathy-causing mutation DNM2-S619L leads to defective tubulation in vitro and in developing zebrafish — Supplementary Material 

# The myopathy-causing mutation DNM2-S619L leads to defective tubulation *in vitro* and in developing zebrafish

## DMM012286 Supplementary Material

**Files in this Data Supplement:**

- **Supplementary Material PDF**
