# Supplementary material for: The myopathy-causing mutation DNM2-S619L leads to defective tubulation in vitro and in developing zebrafish
Source: Dis Model Mech. 2013 Oct 17;7(1):157–61. doi: 10.1242/dmm.012286 (PMC3882057; doi:10.1242/dmm.012286)
Supplement: Supplementary Material [file supp_012286_DMM012286.pdf]

SUPPLEMENTAL MATERIAL

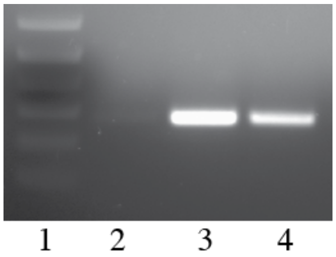

Supplemental Figure 1: Confirmation of DNM2 RNA expression

Embryos were injected with RNA made from either wild type human dynamin-2 cDNA (WT DNM2) or dynamin-2 S619L cDNA (DNM2-S619L) at the 1 cell stage. RNA was extracted at 2 days post fertilization and reverse transcribed with random primers. DNM2 expression was confirmed by PCR with primers that recognize both DNM2 constructs. Lanes: 1- molecular weight standard, 2 – uninjected control, 3- WT DNM2, 4- DNM2-S619L.

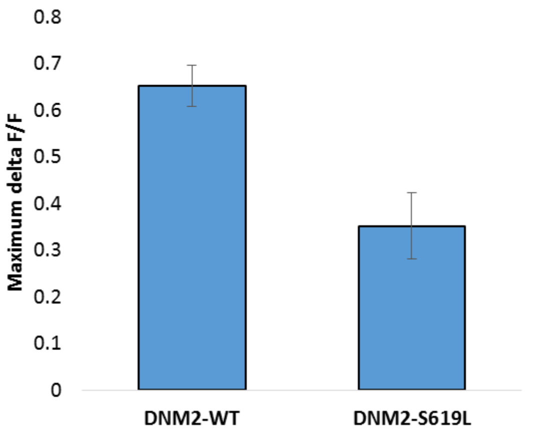

Supplemental Figure 2: Quantification of calcium intensity in DNM2 larval muscle

Fluorescent intensity (deltaF/F) was measured during spontaneous contractions in 24 hpf DNM2-WT and DNM2-S619L larvae injected with GCaMP. The maximum fluorescent intensity within each recording period was identified, and DNM2-S619L larvae displayed a significant decrease in maximum fluorescent intensity as compared with DNM2-WT larvae (DNM2-WT, n=6; DNM2-S619L, n=5; $p<0.004$, Student's t-test).
